# Supplementary material for: Identification and functional analysis of non-coding regulatory small RNA FenSr3 in Bacillus amyloliquefaciens LPB-18
Source: PeerJ. 2023 May 15;11:e15236. doi: 10.7717/peerj.15236 (PMC10194069; doi:10.7717/peerj.15236)
Supplement: Supplemental Information 4 [file peerj-11-15236-s004.zip › KO/CK-vs-T1_map/map05166.html]

KEGG PATHWAY: Human T-cell leukemia virus 1 infection - Reference pathway


|  |  |
| --- | --- |
| **Human T-cell leukemia virus 1 infection - Reference pathway** |  |

[
Pathway menu
| Organism menu
| Pathway entry
| Show description
| User data mapping
]

Human T-cell leukemia virus type 1 (HTLV-1) is a pathogenic retrovirus that is associated with adult T-cell leukemia/lymphoma (ATL). It is also strongly implicated in non-neoplastic chronic inflammatory diseases such as HTLV-1-associated myelopathy/tropical spastic paraparesis (HAM/TSP). Expression of Tax, a viral regulatory protein is critical to the pathogenesis. Tax is a transcriptional co-factor that interfere several signaling pathways related to anti-apoptosis or cell proliferation. The modulation of the signaling by Tax involve its binding to transcription factors like CREB/ATF, NF-kappa B, SRF, and NFAT.

|  |  |  |
| --- | --- | --- |
| Reference pathway | 184% 150% 122% 100% 82% 67% 55% | 图片下载 |
